# Electronic Health Use in the European Union and the Effect of Multimorbidity: Cross-Sectional Survey

**DOI:** 10.2196/jmir.7299

**Published:** 2018-05-03

**Authors:** Francisco Lupiáñez-Villanueva, Dimitra Anastasiadou, Cristiano Codagnone, Roberto Nuño-Solinís, Maria Begona Garcia-Zapirain Soto

**Affiliations:** ^1^ Estudís de Ciencies de la Informació i la Comunicació Universitat Oberta de Catalunya Barcelona Spain; ^2^ Università degli Studi di Milano Milano Italy; ^3^ Deusto Business School Health University of Deusto Bilbao Spain; ^4^ eVIDA Research Group Facultad de Ingeniería University of Deusto Bilbao Spain; ^5^ Facultad de Ingeniería Universidad de Deusto Bilbao Spain

**Keywords:** multimorbidity, eHealth, adoption, Europe

## Abstract

**Background:**

Multimorbidity is becoming increasingly common and is a leading challenge currently faced by societies with aging populations. The presence of multimorbidity requires patients to coordinate, understand, and use the information obtained from different health care professionals, while simultaneously striving to distinguish the symptoms of different diseases and self-manage their sometimes conflicting health problems. Electronic health (eHealth) tools provide a means to disseminate health information and education for both patients and health professionals and hold promise for more efficient and cost-effective care processes.

**Objective:**

The aim of this study was to analyze the use of eHealth tools, taking into account the citizens’ sociodemographic and clinical characteristics, and above all, the presence of multimorbidity.

**Methods:**

Cross-sectional and exploratory research was conducted using online survey data from July 2011 to August 2011. Participants included a total of 14,000 citizens from 14 European countries aged 16 to 74 years, who had used an eHealth tool in the past 3 months. The variables studied were sociodemographic variables of the participants, the questionnaire items assessing the frequency of using eHealth tools, the degree of morbidity, and the eHealth adoption gradient. Chi-square tests were conducted to examine the relationship between the sociodemographic and clinical variables of participants and the group the participants were assigned to according to their frequency of eHealth use (eHealth user group). A one-way analysis of variance (ANOVA) allowed for assessing the differences in the eHealth adoption gradient average between different groups of individuals according to their morbidity level. A two-way between-groups ANOVA was performed to explore the effects of multimorbidity and age group on the eHealth adoption gradient.

**Results:**

According to the eHealth adoption gradient, most participants (68.15%, 9541/14,000) were labeled as rare users, with the majority of them (55.1%, 508/921) being in the age range of 25 to 54 years, with upper secondary education (50.3%, 464/921), currently employed (49.3%, 454/921), and living in medium-sized cities (40.7%, 375/921). Results of the one-way ANOVA showed that the number of health problems significantly affected the use of eHealth tools (*F*_2,13996_=11.584; *P*<.001). The two-way ANOVA demonstrated that there was a statistically significant interaction between the effects of age and number of health problems on the eHealth adoption gradient (*F*_4,11991_=7.936; *P*<.001).

**Conclusions:**

The eHealth adoption gradient has proven to be a reliable way to measure different aspects of eHealth use. Multimorbidity is associated with a more intense use of eHealth, with younger Internet users using new technologies for health purposes more frequently than older groups with the same level of morbidity. These findings suggest the need to consider different strategies aimed at making eHealth tools more sensitive to the characteristics of older populations to reduce digital disadvantages.

## Introduction

### Multimorbidity

Multimorbidity is a growing phenomenon in ageing societies that is especially prevalent among older age groups [1-3]. Although no definition has been uniformly applied across studies, there seems to be a clear association between multimorbidity and lower quality of life (QoL), functional decline, worse health care outcomes (including adverse drug effects, rehospitalization, and mortality), increased use of health care resources, and higher health care costs [3-5]. From a clinical perspective, multimorbidity compounds the complexity of managing chronic diseases. Clinicians, in particular, are faced with a lack of a common theoretical background and guidance on the best care pathways for patients with multimorbidity [6]. In fact, clinical guidelines rarely address comorbidities, and clinical trials often exclude older patients with comorbidities [7,8]. Patients with multimorbidity struggle to make sense of and use the information obtained from different health care professionals and to distinguish between the symptoms of different diseases [9]. Both in terms of service quality and the economic impact, the increasing prevalence of multimorbidity among ageing population represents one of the key health care challenges European countries are currently facing [9-12].

### Electronic Health Tools for Patients With Multimorbidity

Multimorbidity requires, among other things, that both patients and professionals effectively coordinate, synthesize, and reconcile health information from multiple providers and about different conditions [9]. In this respect, electronic health (eHealth) tools have been recommended worldwide to support this type of patient [11,12], as they can improve patient engagement and health outcomes [13,14]. For example, at the European Union level, the European Commission launched a partnership initiative in 2012 (European Innovation Partnership on Active and Healthy Ageing) that aims to join forces between European partners in the development of solutions for active and healthy ageing. International chronic care models of reference also recommend the exploitation of the potential benefits of eHealth tools for the self-management of chronic conditions, emphasizing the need to link informed and actively engaged patients with proactive and prepared health care teams; while also shifting from disease-centered models to patient-centered ones [9,15-17]. In fact, the systematic review by Jong and colleagues [18] revealed improvements in both adherence and health outcomes among chronically ill patients who were using Internet-based self-care techniques, although in other studies the benefits of implementing these applications are still to be proven [19].

To fully understand the potential of eHealth tools for patients with multimorbidity, it is useful to explore how their current usage may or may not differ between Internet users with and without chronic conditions. Results from our scoping review of studies, which aimed to estimate the effect of multimorbidity on the use of eHealth tools, seemed to point to an increasing use of them among people with comorbidities. Patients with a higher comorbidity score were more likely to use the eHealth tools available to them for ordering prescription refills, scheduling appointments, and asking medical or prescription drug related questions [20], as well as for searching, gathering, and sharing online health information with their doctor [21,22]. After controlling for education, sex, and age, Wagner and colleagues [16] found that people with depression, or three or more chronic conditions, were more likely than other chronically ill individuals to use the Internet to search for health information. In addition, qualitative research by Zulman and colleagues [9] revealed the challenges faced by people with comorbidities, including the need to manage a high volume of information from multiple providers, coordinate and synthesize this information and sometimes serve as experts, activities that were found to be associated with emotional distress. Similarly, in a study on the adoption of a personal health record (PHR) [23], patients with multiple chronic conditions were found to have a higher likelihood (25% more likely) of adopting the PHR to track their health condition, compared with those without the selected comorbidities. They were also found to make more intensive use of it, in that they were more likely to use the secure messaging functionality than other patients. Finally, the recent policy brief by Barbabella and colleagues (2016) [11] synthesized the available evidence related to the implementation, benefits, and policies regarding the adoption of eHealth tools for people with multimorbidity in Europe. Authors concluded that eHealth tools for this specific population can significantly improve their health status and QoL through increased integration, personalization, quality, and accessibility of care.

Although the use of eHealth tools may represent an avenue for improving QoL and the capacity to manage the diseases among people with multiple chronic conditions, the concern of health inequality arises in relation to the “digital divide.” If socioeconomic inequalities across different demographic groups (eg, ethnicity, socioeconomic status, age, and gender) determine access to the Internet and the skills to use it effectively, then these may compound health inequality [24]. With respect to this specific dimension, the available empirical evidence is limited and inconclusive. Contradictory findings were reported in studies with samples of veterans [25,26], in which poor health status and some sociodemographic variables, but not comorbidities, were associated with greater eHealth use. The “Pew Research Center” [27,28] reported that people with chronic conditions were significantly less likely than other adults to have Internet access. However, once online, they may more regularly take advantage of the benefits of the health information offered.

### Objective

Within the context of an online survey previously developed and administered in the framework of the Joint Research Centre/Institute for Prospective Technological Studies (JRC/IPTS) “Strategic Intelligence Monitor on Personal Health Systems” (SIMPHS) project in 14 European countries [29], a secondary analysis of the information collected in the study was conducted to assess the use of eHealth tools among the Internet users, with a specific focus on individuals that self-reported multimorbidity conditions. In light of previous research, our first hypothesis was that multimorbidity would lead to greater usage of eHealth tools as a result of more complicated health care needs, a higher likelihood of suffering from adverse events related to their conditions, and greater uncertainty about the effects of treatments on people with multiple conditions. As chronic diseases are often associated with sociodemographic characteristics (ie, advanced age, lower education, and income) and with lower Internet access [30,31], the second hypothesis tested was that when controlling for participants’ age, the opposite trend would be expected: the presence of multiple conditions would be associated with lower usage of eHealth tools. We also expected a gender impact on use of new technologies for health purposes, with men more likely to utilize these tools than women.

## Methods

### Recruitment

This study was cross-sectional and exploratory and used internationally representative survey data from the JRC/SIMPHS project [29]. The target population was composed of citizens aged 16 to 74 years, who had used the Internet in the past 3 months. The survey was carried out from July 2011 to August 2011 and consisted of a total panel of 14,000 interviews from 14 European countries. The participating countries were as follows: Austria, Belgium, Germany, Denmark, Estonia, Finland, France, Italy, Netherlands, Sweden, Slovenia, Slovakia, Spain, and the United Kingdom. It is important to note that as the surveyed population comprised individuals who were already using the Internet, they may be more advanced users than individuals in the general population.

### Survey Instruments and Definition of Variables

First, information concerning the participants’ sociodemographic and health characteristics was collected. Second, the study also collected information regarding eHealth tools use by focusing on the responses that interviewees provided to the question beginning with, “Regarding health, wellness and the Internet, how often have you...?” Individuals were asked about 23 activities that were carried out using eHealth tools. Items were answered on a 5-point Likert scale ranging from “never” to “every day or almost every day.” A previous pilot study based on 231 interviews demonstrated the high internal consistency of the 23 items, which had a Cronbach alpha value of .96 [29].

It is important to note that the word “Internet” was used instead of “eHealth tools” to facilitate better understanding of the questionnaire by the respondents. However, each one of the 23 items of the questionnaire reflected different eHealth tools, delivered or enhanced through the Internet (eg, “Looked for information about a physical illness or condition that you or someone you know has”) and other related technologies such as mobile apps or wearable devices (eg, “...Used a health or wellness app on your mobile phone” and “...Used devices to transmit clinical information, received alarms, followed-up about your health anytime, anywhere”); online platforms (eg, “...Made, canceled, or changed an appointment with your family doctor, specialist or other health professionals online”); email (eg, “...Used email to communicate with a doctor's office”); or videoconference (eg, “...Made an online consultation through videoconference with your doctor or nurse”).

Next, two variables were constructed for the purpose of this study; the first one represents the individual’s degree of morbidity (and multimorbidity), and the second one is an eHealth adoption gradient that has been constructed as a composite indicator of the frequency of use of the 23 activities carried out via eHealth tools.

The measure of multimorbidity was based on self-reported data and defined according to the number of health problems reported by the individual. To explore the effect of multimorbidity on eHealth use, two cut-off points were established: having two of these health problems and having three or more health problems. Although definitions of multimorbidity used in the literature vary widely, disease counting is a very common strategy. Furthermore, the cut-off points followed in this study are commonly used and accepted in the literature. That is, the cut-off of two or more chronic conditions appears to be the norm in multimorbidity prevalence studies in the general population [3], whereas a cut-off point of three or more chronic conditions may also be used in primary care settings [1,32]. Regarding the number of chronic conditions in the list of reference, 13 conditions were included, which fulfils the recommendation for a minimum of 12 conditions proposed by Fortin and colleagues [1]. The number and type of conditions considered when using a disease-counting approach were the factors found to be more critical when comparing prevalence studies on multimorbidity, and the specific list of conditions was taken from a question on reasons for long-term treatment included in a Eurobarometer survey [33]. The specific conditions were as follows: (1) chronic anxiety or depression; (2) peptic ulcer (gastric or duodenal ulcer); (3) stroke, cerebral hemorrhage; (4) osteoporosis; (5) chronic bronchitis, emphysema; (6) migraine or frequent headaches; (7) cataract; (8) cancer; (9) long-standing troubles with your muscles, bones, and joints (rheumatism, arthritis); (10) hypertension (high blood pressure); (11) asthma; (12) an allergy; and (13) diabetes.

To measure the degree of adoption of eHealth tools by the Internet users, we created a composite indicator. As defined by the Organisation for Economic Co-operation and Development/JRC handbook (2008) [34], “a composite indicator is formed when individual indicators are compiled into a single index on the basis of an underlying model. The composite indicator should ideally measure multidimensional concepts which cannot be captured by a single indicator.” Therefore, the construction of this composite indicator aimed to encompass a wide range of eHealth tools following the eHealth global definition suggested by Eysenbach (2001) [35] and supported by Pagliari and colleagues (2005) [36]. We have titled this composite indicator an “eHealth adoption gradient” designed to capture the degree of adoption (ie, usage) of eHealth tools by Internet users and conceptualized as a continuous variable ranging from 0 “no adoption” (no usage) to 1 “frequent usage.”

### Statistical Analysis

The statistical software package SPSS 15.0 (IBM Corp) for Windows was used [37]. To identify the level of multimorbidity, two cut-off points were established: having two of these health problems and having three or more health problems.

For the construction of the composite indicator called “eHealth adoption gradient,” we followed the subsequent steps: first, an analysis was performed on the frequencies of the 23 activities carried out using eHealth tools. Second, to confirm the overlapping content of these activities, the means and their significant correlations were checked. Then, a factor analysis was carried out for data reduction purposes, so as to summarize the information related to the 23 activities reported in the questionnaire asking how often they used the Internet for a list of health-related purposes into a smaller number of unobservable factors (latent variables or constructs). The parameters of these linear functions are referred to as factor loadings. An analysis of the correlation matrix (Kaiser-Meyer-Olkin and Bartlett’s test of sphericity) was carried out to check that the correlation matrixes were factorable. Data reduction was carried out by principal components analysis using the varimax option to identify possible underlying dimensions. Third, a careful and transparent definition of weights was performed, squaring and normalizing the estimated factor loadings from the factor analysis. The squared factor loadings represent the proportion of the total unit variance of a base variable that is explained by a factor. The resulting score by subdimension can be aggregated into the summary indicator of the dimension according to its relative contribution to the explanation of the overall variance of all factors.

The eHealth adoption gradient (composite indicator) was then compared between different groups of individuals according to their level of morbidity (no health problem, one health problem, or two or more health problems) using a one-way analysis of variance (ANOVA). In addition, a two-way between-groups ANOVA was performed to explore the effects of multimorbidity group and age group (16-24, 25-54, and 55-74 years) on the eHealth adoption gradient, followed by post hoc Tukey’s honest significant difference (HSD) tests. Gender was also included as a covariate in an analysis of covariance (ANCOVA) of comparisons between age group and morbidity levels. Finally, a two-way between-groups ANOVA was performed to explore the effects of age group and the receival (or not) long-term medical treatment group on the eHealth adoption gradient, followed by post hoc Tukey’s HSD tests. All statistical tests were conducted with a criterion for significance at *P*<.05. All bivariate and multivariate analyses were weighted.

## Results

### Frequency and Type of Electronic Health Use

The list of items and the frequency of each response are shown in Table 1. With regard to the activities carried out using eHealth tools, 81.60% of the surveyed individuals (11,424/14,000) used the Internet at least once a month to look for information about a physical illness or condition, and 75.30% (10,542/14,000) of the individuals used eHealth tools for wellness or lifestyle. Nevertheless, in other eHealth tools such as mobile apps or wearable devices (eg, “...Used a health or wellness app on your mobile phone” and “...Used devices to transmit clinical information, received alarms, followed-up about your health anytime, anywhere”), online platforms (eg, “...Made, canceled, or changed an appointment with your family doctor, specialist or other health professionals online”), or emails (eg, “...Used email to communicate with a doctor’s office”), we observed less frequent use, with the percentage of the surveyed individuals who had never engaged in the other 21 possible uses varying between 15.82% (2215/14,000) and 87.43% (12,240/14,000). See Table 1 for frequency rates of responses.

### Further Examination of the Electronic Health Adoption Construct

The factor analysis performed helped us to summarize the 23 activities in two distinctive components, which together explained 73% of the variance: (1) a factor composed of 13 items assessing the engagement in common activities related to “information and communication” (explaining 49% of the variance), which represented a basic usage of eHealth tools and (2) a factor formed by 10 items, which evaluated the use of eHealth tools related to “services and devices” (explaining 31% of the variance), which represented a more advanced type of usage. The results of this analysis are presented in Table 2.

These two factors were then used to construct a composite indicator as reported in the statistical analysis section, the “eHealth adoption gradient,” which was used as a continuous variable for further comparisons performed in this study.

Then, after applying cluster analysis to the eHealth adoption index, a three-cluster solution emerged (two recommended cut-off points at 0.38 and 0.81), allowing us to divide participants into three groups (rare users, normal users, and super users) depending on how frequently they used eHealth tools [38].

According to the cut-off points recommended for the eHealth adoption gradient, 9,541 of the 14,000 participants (68.15%) were considered as rare users. Participants who sometimes used the Internet for health purposes during the week or month were classified as normal users (3538/14,000; 25.27%), and those who engaged in health-related behaviors online even more frequently were considered super users (921/14,000; 6.57%).

**Table 1:** Frequency rates of responses on electronic health (eHealth) uses.

Responses	Never, n (%)	Less than once a month, n (%)	At least once a month (but not every week), n (%)	At least once a week (but not every day), n (%)	Every day or almost every day, n (%)
Looked for information about a physical illness or condition that you or someone you know has	2147 (15.82)	5641 (41.57)	3505 (25.83)	1757 (12.95)	519 (3.82)
Looked for information about wellness or lifestyle	2930 (21.75)	4614 (34.26)	3419 (25.38)	1913 (14.20)	593 (4.40)
Participated in an online support group for people who are concerned about the same health or medical issue	8445 (68.50)	1758 (14.26)	1114 (9.04)	728 (5.90)	284 (2.30)
Participated in social networking sites (SNSs) talking about health and wellness	8121 (65.13)	1987 (15.94)	1197 (9.60)	817 (6.55)	347 (2.78)
Used email or went to a website to communicate with a doctor’s office	8045 66.43)	2015 (16.64)	1112 (9.18)	632 (5.22)	306 (2.53)
Clicked on a health or medical website’s privacy policy to read about how the site uses personal information	7291 (59.47)	2421 (19.75)	1323 (10.79)	864 (7.05)	361 (2.94)
Described a medical condition or problem online to get advice from an online doctor	8538 (69.91)	1796 (14.71)	966 (7.91)	614 (5.03)	299 (2.45)
Described a medical condition or problem online in order to get advice from other online users	8133 (64.81)	2199 (17.52)	1198 (9.55)	730 (5.82)	289 (2.30)
Kept a health website “bookmarked” or saved as a “favorite place,” so you can go back to it regularly	6235 (48.72)	2763 (21.59)	1878 (14.68)	1327 (10.37)	594 (4.64)
Looked to see what company or organization is providing the advice or information that appears on a health website	6196 (48.53)	3293 (25.79)	1930 (15.12)	981 (7.68)	367 (2.87)
Looked for information about a mental health issue such as depression or anxiety	6482 (50.76)	3185 (24.94)	1691 (13.24)	980 (7.67)	433 (3.39)
Disclosed medical information on SNSs	9189 (75.64)	1175 (9.67)	899 (7.40)	596 (4.91)	290 (2.39)
Disclosed medical information on websites to share pictures, videos, movies, etc.	9325 (77.46)	1023 (8.50)	801 (6.65)	626 (5.20)	264 (2.19)
Made, canceled, or changed an appointment with your family doctor, specialist, or other health professionals online	9203 (70.00)	2236 (17.01)	904 (6.88)	520 (3.95)	285 (2.17)
Sent or received an email from your doctor, nurse, or health care organization	9554 (72.89)	2049 (15.63)	772 (5.89)	521 (3.97)	211 (1.61)
Made an online consultation through videoconference with your doctor or nurse	11,044 (87.43)	492 (3.89)	528 (4.18)	368 (2.91)	200 (1.58)
Received the results of your clinical or medical test online	10,476 (81.23)	1160 (8.99)	612 (4.75)	457 (3.54)	192 (1.49)
Accessed or uploaded your medical information or health record through an Internet protocol	10,801 (85.21)	661 (5.21)	589 (4.65)	438 (3.46)	187 (1.48)
Accessed or uploaded your medical information or health record through an Internet application provided by your health care organization	10,703 (84.31)	756 (5.96)	596 (4.69)	446 (3.51)	194 (1.53)
Used a game console to play games related with your health or your wellness	10,054 (77.90)	1191 (9.23)	855 (6.62)	569 (4.41)	237 (1.84)
Used a health or wellness app on your mobile phone	10,728 (82.25)	891 (6.83)	686 (5.26)	537 (4.12)	201 (1.54)
Used devices to transmit clinical information, received alarms, followed-up about your health anytime, anywhere	10,299 (79.28)	1069 (8.23)	785 (6.04)	560 (4.31)	278 (2.14)
Received any message about health promotion or health prevention	8575 (64.57)	2312 (17.41)	1292 (9.73)	765 (5.76)	337 (2.54)

**Table 2:** Results of factor analyses of electronic health (eHealth) uses. Rotated components matrix; sampling method: factor analysis by main components; rotation method: varimax with Kaiser-Meyer-Olkin 0.984; Bartlett’s test of sphericity *P*<.001; convergence in three itineration; minimum eigenvalue 1.

Electronic health uses	Factor 1: services and devices		Factor 2: information and communication	
	Factor loadings^a^	Weights of variables in factor^b^	Factor loadings^a^	Weights of variables in factor^b^
Made an online consultation through videoconference with your doctor or nurse	0.88	0.09	0.33	0.01
Accessed or uploaded your medical information or health record through an Internet protocol	0.87	0.08	0.35	0.02
Accessed or uploaded your medical information or health record through an Internet application provided by your health care organization	0.86	0.08	0.35	0.02
Received online the results of your clinical or medical test	0.85	0.08	0.34	0.01
Used a health or wellness app on your mobile phone	0.81	0.07	0.34	0.02
Sent or received an email from your doctor, nurse, or health care organization	0.78	0.07	0.37	0.02
Used devices to transmit clinical information, received alarms, follow-up about your health anytime, anywhere	0.78	0.07	0.30	0.01
Made, canceled, or changed an appointment with your family doctor, specialist, or other health professionals online	0.77	0.07	0.35	0.025
Used a game console to play games related with your health or your wellness	0.76	0.06	0.31	0.01
Received any message about health promotion and/or health prevention	0.63	0.04	0.42	0.02
Looked for information about a physical illness or condition that you or someone you know has	0.17	0.01	0.78	0.08
Looked to see what company or organization is providing the advice or information that appears on a health website	0.32	0.01	0.77	0.08
Looked for information about wellness or lifestyle	0.16	0.01	0.75	0.07
Participated in social networking sites (SNSs) talking about health and wellness	0.46	0.02	0.74	0.07
Described a medical condition or problem online to get advice from other online users	0.50	0.03	0.72	0.07
Kept a health web site “bookmarked” or saved as a “favorite place,” so you can go back to it regularly	0.28	0.01	0.71	0.07
Looked for information about a mental health issue such as depression or anxiety	0.35	0.01	0.71	0.07
Participated in an online support group for people who are concerned about the same health or medical issue	0.50	0.03	0.71	0.06
Clicked on a health or medical website’s privacy policy to read about how the site uses personal information	0.42	0.02	0.71	0.06
Described a medical condition or problem online to get advice from an online doctor	0.54	0.03	0.69	0.06
Disclosed medical information on SNSs	0.57	0.04	0.67	0.06
Disclosed medical information on websites to share pictures, videos, movies, etc.	0.58	0.04	0.66	0.06
Used email or gone to a website to communicate with a doctor’s office	0.54	0.03	0.64	0.05
Percentage of variance explained (%)	31		49	

^a^Based on rotated component matrix.

^b^Normalized squared factor loadings.

### Sociodemographic and Clinical Data of Participants by User Group

Table 3 shows the descriptive statistics of the sociodemographic and clinical variables by eHealth user groups (rare users, normal users, and super users). Among the super users, 55.1% (508/921) were aged 25 to 54 years, 50.3% (464/921) had upper secondary education, 49.3% (454/921) were employed, and 40.7% (375/921) lived in medium-sized cities. Furthermore, 61.3% (565/921) also reported no health problem, a significantly higher percentage than the 47.76% registered among the normal users (1690/3538) and 55.73% (5317/9540) among the rare users (χ^2^_2_=156.8; *P*<.001). When examining the most frequently reported health problems, 3221 of the 9541 rare users (1422 of the 3538 normal users, 40.19%), and 257 of the 921 of super users (27.9%) reported allergy problems (χ^2^_2_=68.9; *P*<.001). In turn, 2728 of the 9541 rare users (28.59%), 1287 of the 3538 normal users (36.37%), and 227 of the 921 super users (24.6%) reported migraine or frequent headaches (χ^2^_2_=88.9; *P*<.001). In addition, 2110 of the 9541 rare users (22.11%), 990 of the 3538 normal users (27.98%), and 213 of the 921 super users (23.1%) reported strong, long-standing troubles with muscles and bones. Finally, 1765 of the 9541 rare users (18.49%), 908 of the 3538 normal users (25.66%), and 123 of the 921 super users (13.3%) reported chronic anxiety or depression (χ^2^_2_=110.0; *P*<.001).

### Information and Communication Technology for Health by Morbidity Levels

Results of the one-way ANOVA showed that the number of health problems had a significant effect on the use of eHealth use (*F*_2,13996_=11.584; *P*<.001). Post hoc comparisons using the Tukey HSD test indicated that participants with two or more health problems used new technologies for health significantly more (mean= 0.36; SD=0.23) compared with participants with no health problem (mean=0.33, SD=0.27) and participants with one health problem (mean=0.34, SD=0.25). However, there were no significant differences between participants with no health problem and those with one health problem on their use of eHealth.

A similar pattern was observed in the frequency of eHealth use with the series of one-way ANOVAs that were carried out to examine effect of health status group (no health problem, one health problem, and two or more health problems) on the mean scores of the 23 items on the questionnaire (Table 1). For example, in the question “How often have you looked for information about a physical illness or condition that you or someone you know has,” participants with two or more health problems used eHealth significantly more to access health-related information (mean=1.59, SD=1.06) than participants with no health problem (mean=1.22, SD=1.04) and participants with one health problem (mean=1.36, SD=0.98; *F*_2,13996_=164.127; *P*<.001).

**Table 3:** Sociodemographic and clinical data of participants by group of electronic health (eHealth) user.

Sociodemographic and clinical data		Rare users (N=9541), n (%)	Normal users (N=3538), n (%)	Super users (N=921), n (%)	Chi-square (degrees of freedom); *P* value
**Age groups (years)**					
	16-24	1645 (17.24)	932 ( 26.35)	200 (21.6)	209.1 (4); <.001
	25-54	6031 (63.21)	2169 (61.32)	508 (55.1)	
	55-74	1861 (19.54)	436 (12.32)	214 (23.2)	
**Gender**					
	Male	4896 (51.31)	1859 (52.54)	456 (49.4)	3213 (2); .20
	Female	4645 (48.68)	1679 (47.45)	466 (50.6)	
**Educational level**					
	Primary or lower secondary education (international standard classification of education, ISCED 3 or 4)	1415 (14.83)	529 (14.83)	184 (19.9)	45.2 (2); <.001
	Upper secondary education (ISCED 3 or 4)	4306 (45.13)	1670 (47.18)	464 (50.3)	
	Tertiary education (ISCED 5 or 6)	3819 (40.03)	1340 (37.86)	274 (29.8)	
**Occupation**					
	Employed or self-employed (including family workers)	5644 (59.16)	2091 (59.10)	454 (49.3)	131.2 (6); <.001
	Unemployed	892 (9.35)	315 (8.90)	128 (13.9)	
	Student (not in the workforce)	1239 (12.98)	642 (18.14)	126 (13.7)	
	Other not in the workforce (eg, retired or inactive)	1765 (18.50)	490 (13.84)	213 (23.7)	
**Type of locality**					
	Densely populated area (cities and large towns)	3660 (38.36)	1469 (41.52)	338 (36.7)	48.6 (2); <.001
	Intermediate area (towns)	3609 (37.82)	1423 (40.22)	375 (40.7)	
	Thinly populated area (village and rural)	2272 (23.81)	646 (18.25)	208 (22.6)	
**Presence of long-standing illness or health problem**					
	Yes	3819 (40.02)	1473 (41.63)	364 (39.5)	4566 (2); .10
	No	5422 (56.82)	1918 (54.21)	510 (55.4)	
**Long-term medical treatment**					
	Yes	3197 (33.50)	1228 (34.71)	307 (33.3)	2146 (2); .34
	No	6269 (65.70)	2266 (64.05)	596 (64.7)	
**Comorbidities**					
	No health problem	5317 (55.72)	1690 (47.76)	565 (61.3)	156.8 (4); .001
	One health problem	2122 (22.24)	722 (20.4)	167 (18.2)	
	Two or more health problems	2101 (22.02)	1126 (31.82)	189 (20.5)	

**Table 4:** Two-way between-groups analysis of variance of electronic health (eHealth) adoption gradient.

Variable		Mean (SD)			Source of variation	*F* value (degrees of freedom)	*P* value	Partial eta squared
		No health problem	One heath problem	Two or more health problems				
**eHealth adoption gradient (age range, years)**								
	16-24	0.41 (0.30)	0.40 (0.27)	0.39 (0.22)	Age	78.248 (2)	.001	0.013
	25-54	0.32 (0.26)	0.33 (0.23)	0.37 (0.23)	Comorbidities	5.6747.936 (2)	.003	0.001
	55-74	0.28 (0.24)	0.33 (0.26)	0.33 (0.24)	Age x comorbidities	7.936 (2,2)	.001	0.003

In regards to the use of eHealth to participate in online support groups, in response to the question “How often have you participated in an online support group for people who are concerned about the same health or medical issue,” participants with two or more health problems participated in online support groups significantly more frequently (mean=0.62, SD=1.04) than participants with no health problem (mean=0.42, SD=0.92) and participants with one health problem (mean=0.46, SD=0.93; *F*_2,13996_= 61.328; *P*<.001). The post hoc comparisons between individuals who did not report any health problem and those with one health problem were not significant for either of the two items described above.

### The Electronic Health Adoption Gradient by Morbidity Level and Age Group

A two-way ANOVA was conducted to examine the effect of age and morbidity level on the eHealth adoption gradient (Table 4). There was a statistically significant interaction between the effects of age and number of health problems on the eHealth adoption gradient (*F*_4,11991_=7.936; *P*<.001. Simple main effects analysis showed that people aged 16 to 24 years used new technologies for health purposes significantly more than people aged 25 to 54 years (*P*<.001) and people aged 55 to 74 years (*P*<.001) with no health problem, and also that people aged 25 to 54 years with no health problem used eHealth tools significantly more than people aged 55 to 74 years (*P*=.003). In addition, people with one health problem aged 16 to 24 years used eHealth tools significantly more than people aged 25 to 54 years (*P*<.001) and people aged 55 to 74 years (*P*<.001), but there were no statistically significant differences in the use of eHealth between people with one health problem aged 25 to 54 years and people aged 55 to 74 years (*P*=.99). Furthermore, people with one or more comorbidities (two or more health problems) aged 16 to 24 years used eHealth tools significantly more than people aged 25 to 54 years (*P*=.007) and people aged 55 to 74 years (*P*<.001), and there were also statistically significant differences in the use of eHealth between people with one or more comorbidities aged 25 to 54 years and people aged 55 to 74 years *(P*<.001).

To contrast these results, a comparison of the average eHealth adoption gradient was also performed on other health status variables included in the questionnaire of reference for this analysis. In particular, a two-way ANOVA was performed to examine the effect of age and the response to a question regarding whether the individual was receiving long-term medical treatment. There were main effects of age (*F*_2,11587_=8.350; *P*<.001) and whether or not they were receiving long-term medical treatment (*F*_2,11587_=11.160; *P*<.001) on the use of eHealth adoption gradient, but there was not a significant interaction between the aforementioned variables (*F*_4,11587_=1.729; *P*=.14). Simple main effects analyses showed that the frequency of use of eHealth was greater for younger participants (16-24 > 25-54 > 55-74 years), with *P*<.001 for the three comparisons. In addition, people undergoing long-term medical treatment used eHealth tools significantly more than people without treatment (*P*<.001). The results follow a different pattern from the one described previously, with those individuals who reported long-term medical treatment using eHealth tools more frequently, independent of the age group to which they belonged.

### Gender as a Covariate

When gender was included as a covariate in an ANCOVA of comparisons between age group and morbidity levels, results showed that gender had no significant effect on the eHealth adoption gradient (*F*_1,11990_=0.748; *P*=.39).

## Discussion

### Principal Findings

The aim of this study was to explore the frequency of use of eHealth tools among a representative sample of European Internet users, taking into account their sociodemographic and clinical characteristics. We also examined the effect of multimorbidity on eHealth use. There was a positive and significant association between the number of health problems and the use of eHealth. An age-based digital divide was also observed, with individuals in younger age groups reporting a higher use of eHealth than older groups with the same level of morbidity. These findings add support to previous research, which has suggested that people with multiple conditions may demonstrate increased use of the available eHealth solutions. However, the full potential of eHealth tools for this population in particular has not yet been fully explored. There are only a limited number of studies and policies that have addressed people with multiple chronic conditions because of the fact that the disease-oriented approach still influences clinical research and health care organizations [11]. Therefore, it is important to move from a disease-centered to a patient-centered model [9] and increase our understanding of the experiences, specific needs, and challenges that may be faced by this population to improve the efficiency and quality of the existing eHealth services and encourage the implementation of new ones [10,11].

### Main Findings

The eHealth adoption gradient proved to be a reliable way to measure different aspects of eHealth use [29]. A striking finding was that although most of the users went online to look for health information at least once a month, the majority of participants were classified as rare users, and only 921 out of the 14,000 total participants (6.57%) were super users. A closer exploration of the sociodemographic and clinical characteristics of the most frequent eHealth users reveals that the majority of them were aged 25 to 54 years and reported having zero health problems. This would suggest that those individuals who most frequently use eHealth do so for prevention, rather than as a reaction to a health concern. In addition, there were not gender differences in the use of the eHealth purposes, which is contrary to previous findings which concluded that females were more likely to look for health information online than males [27,39].

This study shows that multimorbidity, defined as the reporting of two coexisting health problems, is associated with more intense use of eHealth, both for information and communication purposes (eg, information about a physical illness or lifestyle and participation in online support groups), as well as for health-related services and devices (eg, videoconferences, Internet or mHealth apps, games, and mails to or from the general practitioner), a finding that is consistent with previous research [20-22]. It is interesting to note that the use of new technologies for health purposes was greater in questions related to seeking heath information on the Internet, compared with other uses of eHealth, such as participation in support groups, exchange of personal information online, or communication online with medical staff. These findings are similar to the ones reported by Zulman and colleagues (2015) [9]. Issues related to security and confidentiality of online personal information may explain the lower use of eHealth for the aforementioned specific purposes, as they are considered barriers for Internet use [27,28].

The existence of an age-based digital divide was observed when we included the age group of eHealth users in a two-way ANOVA. Our results showed that individuals in younger age groups reported greater use of eHealth solutions than older groups with the same level of morbidity. For example, individuals with two or more health problems aged 16 to 24 years used eHealth more frequently than those in the older age groups of 24 to 54 years and 55 to 74 years. Furthermore, healthy people aged 16 to 24 years were more likely to engage in eHealth activities than those aged 25 to 54 years and 55 to 74 years. It is also worth noting that the post hoc comparison between individuals who did not report any health problem and those with one health problem was not significant, but this may be because of the limited number of health problems considered (13), which does not preclude the presence of other conditions among those who reported no health problem. In any case, the interest of this exploratory study focuses on the effect of multimorbidity, for which the number of conditions considered should be sufficient, according to Fortin et al (2012) [40]. As expected, in line with the results of previous studies [29,31], the use of eHealth decreased with age in all morbidity groups.

### Limitations

The principal limitation of this study concerns possible selection bias. That is, the surveyed population was already Internet users, and those with chronic illness may also have been less inclined to participate in an online survey or were unable to participate because of fatigue or disability. It would be interesting for future studies to test whether the relationship observed in this study between multimorbidity and eHealth use remains valid in a representative sample of multimorbid patients from the general population. Another limitation of this study is that the population studied only included citizens up to 74 years of age, whereas the prevalence of multimorbidity is particularly high in the oldest age groups, among which use of the eHealth is expected to be lower [30]. In addition, given the absence of a severity scale for the assessment of multimorbidity, it might also be worth exploring different and more complex ways of assessing it in future research. For example, some authors have criticized the simple counting of chronic diseases as oversimplistic, as it does not allow for discriminating between groups, particularly at older ages. Instead, they propose a more holistic approach that considers additional factors such as “emotional and psychological distress, and even existential or spiritual distress, all of which are socially patterned” [41]. Furthermore, self-reporting of chronic diseases may also represent a limitation for our study, as many people need further information and explanation to better understand if they have a specific chronic disease or not. In addition, the large proportion of rare users found in this study poses questions in terms of generalizability of our findings. Finally, the level of motivation and perception of barriers of the participants with multiple conditions were not evaluated in this study. Future research would benefit from identifying potential barriers (eg, low level of digital skills, lack of personalization of eHealth devices, or high perceived costs [6,11]) and assessing variables related to motivation (eg, how can eHealth be used to increase users’ motivation to engage with sustainable lifestyle changes and how people can be motivated to use eHealth solutions? [6]) in this specific population.

### Conclusions

In conclusion, it seems plausible that people with multimorbidity would benefit from new technologies for health-related activities as an additional means of communication. However, multimorbidity affects mostly older people who, in turn, are less likely to ask for support online. Therefore, complementary studies should test and implement new ways of making the already available eHealth solutions more accessible, attractive, and sensitive to the needs of older people to reduce the digital disadvantage which the majority of older adults may experience. For example, comprehensive training and educational campaigns addressing low digital literacy for health purposes for older people affected by multiple conditions and their informal caregivers can be offered. In addition, exploring the association between multimorbidity and eHealth use in a sample of individuals that are more representative of the general population with multimorbidity, including not only Internet users but also nonusers and persons in age groups older than 74 years, would be an interesting area for future research.

## Abbreviations

ANOVA: analysis of variance
ANCOVA: analysis of covariance
eHealth: electronic health
HSD: honest significant difference
IPTS: Institute for Prospective Technological Studies
JRC: Joint Research Centre
PHR: personal health record
QoL: quality of life
SIMPHS: Strategic Intelligence Monitor on Personal Health Systems
